# Supplementary material for: Genome-Wide Analysis of the UGT Gene Family and Identification of Flavonoids in Broussonetia papyrifera
Source: Molecules. 2021 Jun 6;26(11):3449. doi: 10.3390/molecules26113449 (PMC8200958; doi:10.3390/molecules26113449)
Supplement: Supplementary file 1 [file molecules-26-03449-s001.zip › molecules-1203820-supplementary.pdf]

Supplementary Materials: Genome-Wide Analysis of the *UGT* Gene Family and Identification of Flavonoids in *Broussonetia Papyrifera*

Fenfen Wang [1,2], Yalei Su [1,2], Naizhi Chen [1,*] and Shihua Shen [1,*]

[1] Key Laboratory of Plant Resources, Institute of Botany, the Chinese Academy of Sciences, Beijing 100093, China; wangfenfen@ibcas.ac.cn (F.W.); suyalei@ibcas.ac.cn (Y.S.)
[2] University of Chinese Academy of Sciences, Beijing 100049, China
* Correspondence: chennaizhi@ibcas.ac.cn (N.C.); shshen@ibcas.ac.cn (S.S.)

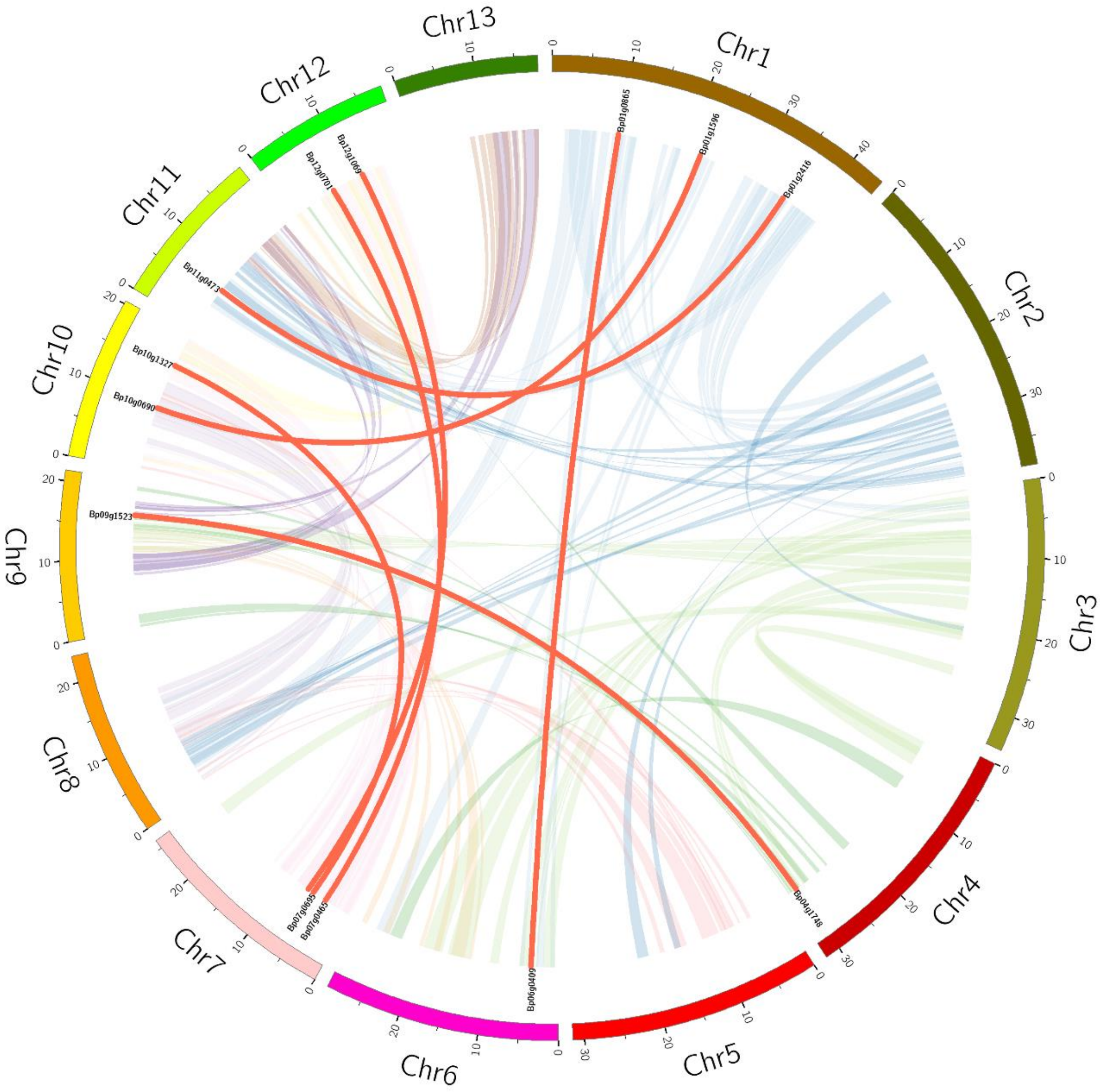

Figure S1. Schematic representations of segmental duplications of the paper mulberry *BpUGTs*. Different color lines indicate all synteny blocks in paper mulberry genome between each chromosome, and the thick red lines indicate duplicated *BpUGT* gene pairs. The chromosome number is indicated at the bottom of each chromosome. Scale bar marked on the chromosome indicating chromosome lengths (Mb).

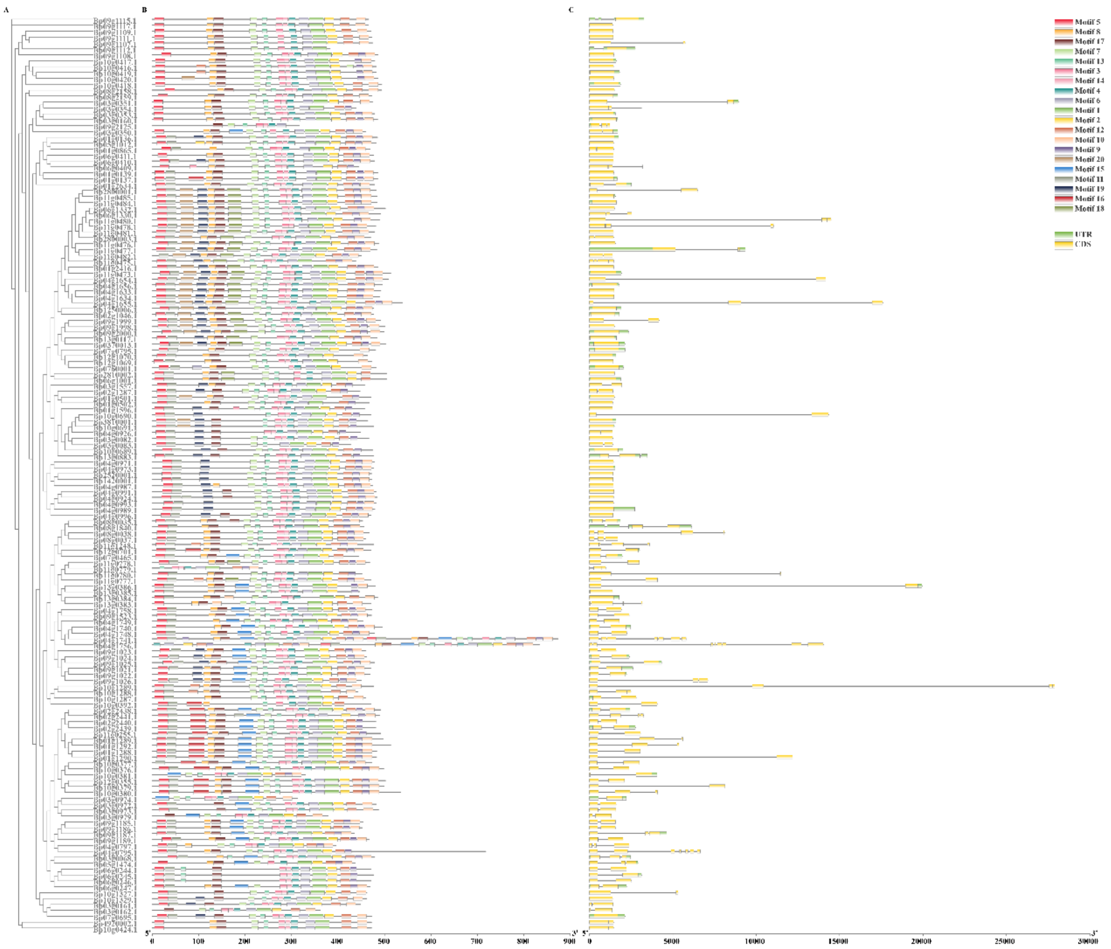

Figure S2. Phylogenetic relationship, gene structure and conserved motif analysis of the *BpUGT* family. (**A**) The conserved UGT domain amino acid sequences of BpUGT proteins were aligned and a ML tree was constructed by MEGA X with 1,000 bootstrap replicates. (**B**) The gene structure of *BpUGTs*. Exons, introns and untranslated regions (UTRs) are indicated by yellow rectangles, black lines and green rectangles, respectively. (**C**) The multiple conserved motifs of BpUGT proteins. Different colored rectangles represent different motifs.

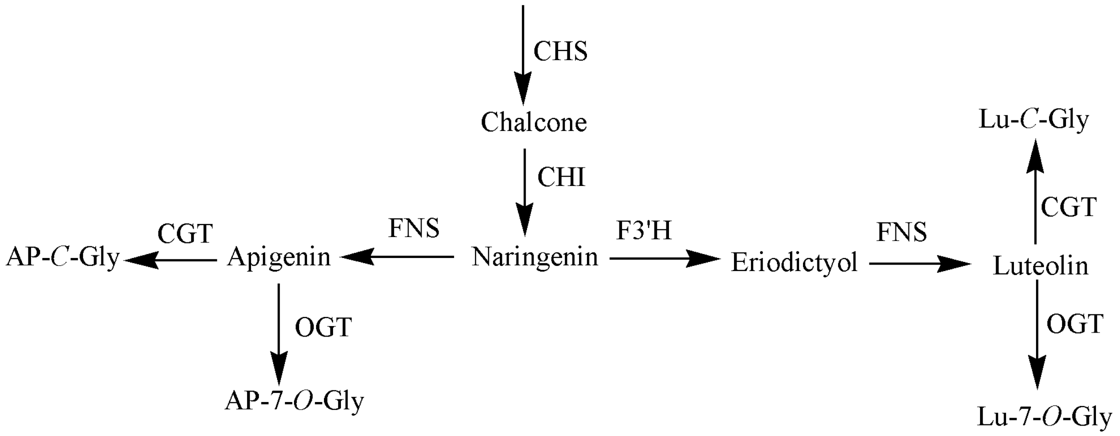

Figure S3. Putative flavonoid *C*-glycosides and *O*-glycosides biosynthesis pathway in paper mulberry. CHS: chalcone synthase; CHI: chalcone isomerase; F2H: flavanone 2-hydroxylase; F3′H: flavanone 3′-hydroxylase; CGT: C-glycosyltransferase; Gly: glycoside; Ap: apigenin; Lu: luteolin.

Table S1: The detailed information of 155 *BpUGTs* identified in paper mulberry.

ID	groups	Chromosome	aa length	CDS（bp）	pI	MW
Bp01g1596.1	A	chr01	457	1371	5.61	51041
Bp3810001.1	A	scaffold_381	464	1392	6.33	51843
Bp10g0689.1	A	chr10	475	1425	6.66	52641
Bp10g0690.1	A	chr10	471	1413	6.48	53155
Bp10g0691.1	A	chr10	477	1431	6.13	53658
Bp04g0926.1	A	chr04	448	1344	5.79	50201
Bp03g0083.1	A	chr03	427	1281	6.65	48326
Bp03g0082.1	A	chr03	466	1398	6	52367
Bp13g0883.1	A	chr13	477	1431	5.38	53218
Bp01g0501.1	A	chr01	471	1413	6.44	53130
Bp01g0502.1	A	chr01	467	1401	5.99	52710
Bp02g1287.1	A	chr02	447	1341	5.48	50151
Bp03g1557.1	A	chr03	458	1374	6.46	52530
Bp0760001.1	B	scaffold_076	482	1446	6.44	53002
Bp12g1069.1	B	chr12	473	1419	5.85	52409
Bp12g1070.1	B	chr12	463	1389	5.69	51173
Bp07g0795.1	B	chr07	481	1443	5.99	52315
Bp02g1046.1	C	chr02	476	1428	6.25	52232
Bp1250006.1	C	scaffold_125	477	1431	6	53370
Bp11g0478.1	D	chr11	481	1443	5.43	53920
Bp11g0480.1	D	chr11	490	1470	5.97	55529
Bp11g0481.1	D	chr11	479	1437	5.94	54052
Bp11g0482.1	D	chr11	450	1350	5.71	50297
Bp11g0484.1	D	chr11	484	1452	5.48	54648
Bp11g0485.1	D	chr11	484	1452	6.13	53760
Bp06g1330.1	D	chr06	491	1473	6.1	54984
Bp06g1332.1	D	chr06	502	1506	6.39	56195
Bp2800001.1	D	scaffold_280	482	1446	6.33	53853
Bp2800003.1	D	scaffold_280	470	1410	5.57	53112
Bp11g0476.1	D	chr11	487	1461	5.91	55000
Bp11g0475.1	D	chr11	438	1314	7.16	49942
Bp11g0477.1	D	chr11	483	1449	5.94	54383
Bp11g0473.1	D	chr11	513	1539	6.23	58326
Bp01g2416.1	D	chr01	486	1458	5.76	54641
Bp04g1633.1	D	chr04	492	1476	5.53	54993
Bp04g1634.1	D	chr04	489	1467	5.19	55142
Bp04g1654.1	D	chr04	508	1524	5.79	57013
Bp04g1655.1	D	chr04	538	1614	6.6	60716
Bp04g1656.1	D	chr04	495	1485	6.02	54381
Bp09g1107.1	E	chr09	474	1422	6.13	52694
Bp09g1108.1	E	chr09	486	1458	5.87	53607
Bp09g1109.1	E	chr09	470	1410	6.34	52352
Bp09g1111.1	E	chr09	473	1419	6.26	52700
Bp09g1112.1	E	chr09	383	1149	5.78	42680
Bp09g1115.1	E	chr09	466	1398	5.51	52067
Bp09g1117.1	E	chr09	465	1395	5.69	51954
Bp4920002.1	E	scaffold_492	472	1416	5.32	52205
Bp10g0424.1	E	chr10	472	1416	5.39	52230

Bp10g0416.1	E	chr10	482	1446	5.48	53281
Bp10g0417.1	E	chr10	479	1437	5.29	53222
Bp10g0418.1	E	chr10	494	1482	5.32	54809
Bp10g0419.1	E	chr10	482	1446	5.94	53416
Bp10g0420.1	E	chr10	485	1455	5.34	53720
Bp08g2158.1	E	chr08	494	1482	5.88	55008
Bp08g2159.1	E	chr08	473	1419	6.18	52844
Bp03g0350.1	E	chr03	460	1380	5.03	51084
Bp03g0351.1	E	chr03	474	1422	6.28	52055
Bp03g0353.1	E	chr03	479	1437	5.82	52651
Bp03g0354.1	E	chr03	439	1317	5.23	48222
Bp03g0160.1	E	chr03	485	1455	5.58	53487
Bp09g2125.1	E	chr09	316	948	8.12	35127
Bp05g1012.1	E	chr05	482	1446	5.38	53207
Bp01g0865.1	E	chr01	468	1404	5.99	51113
Bp01g0136.1	E	chr01	485	1455	5.76	53116
Bp06g0409.1	E	chr06	445	1335	5.79	49606
Bp06g0410.1	E	chr06	478	1434	5.95	53030
Bp06g0411.1	E	chr06	476	1428	6.25	52493
Bp01g2634.1	E	chr01	478	1434	5.31	53177
Bp01g0137.1	E	chr01	485	1455	6.2	52933
Bp01g0139.1	E	chr01	485	1455	5.57	53026
Bp04g0924.1	E	chr04	484	1452	6.54	53277
Bp07g0695.1	E	chr07	472	1416	6.35	51520
Bp03g0161.1	E	chr03	448	1344	5.42	49598
Bp03g0162.1	E	chr03	361	1083	6.35	40618
Bp10g1327.1	E	chr10	462	1386	6.33	51028
Bp10g1329.1	E	chr10	462	1386	5.75	51255
Bp04g0989.1	E	chr04	479	1437	6.29	53320
Bp04g0991.1	E	chr04	482	1446	6.73	53042
Bp04g0993.1	E	chr04	482	1446	6.9	54208
Bp04g0996.1	E	chr04	471	1413	6.07	52988
Bp1420001.1	E	scaffold_142	472	1416	5.68	52924
Bp2520001.1	E	scaffold_252	472	1416	5.68	52864
Bp04g0971.1	E	chr04	478	1434	6.24	53737
Bp04g0973.1	E	chr04	477	1431	7.32	53553
Bp04g0987.1	E	chr04	475	1425	5.85	52953
Bp03g0068.1	F	chr03	479	1437	6.53	52818
Bp05g1474.1	F	chr05	437	1311	5.68	47918
Bp06g0244.1	F	chr06	471	1413	7.66	51429
Bp06g0245.1	F	chr06	477	1431	7.19	52142
Bp06g0246.1	F	chr06	471	1413	7.22	51490
Bp06g0247.1	F	chr06	469	1407	5.52	51481
Bp03g0972.1	G	chr03	482	1446	6.2	54220
Bp03g0973.1	G	chr03	487	1461	5.89	54526
Bp03g0974.1	G	chr03	312	936	7.01	35428
Bp03g0979.1	G	chr03	379	1137	5.46	42446
Bp02g2438.1	G	chr02	492	1476	5.23	55053
Bp02g2439.1	G	chr02	481	1443	5.4	53853
Bp02g2440.1	G	chr02	481	1443	5.08	53880
Bp02g2441.1	G	chr02	489	1467	5.36	54890
Bp11g0255.1	G	chr11	492	1476	5.71	55391

Bp01g1288.1	G	chr01	483	1449	5.09	54431
Bp01g1289.1	G	chr01	497	1491	5.45	55989
Bp01g1290.1	G	chr01	483	1449	5.28	54469
Bp01g1292.1	G	chr01	513	1539	5.71	57904
Bp10g0376.1	G	chr10	498	1494	6.18	55587
Bp10g0377.1	G	chr10	473	1419	5.49	52988
Bp10g0379.1	G	chr10	499	1497	5.25	55904
Bp10g0380.1	G	chr10	534	1602	5.52	59248
Bp10g0381.1	G	chr10	329	987	5.31	37512
Bp12g0355.1	G	chr12	502	1506	5.44	56190
Bp09g1185.1	H	chr09	454	1362	6.44	50560
Bp09g1186.1	H	chr09	452	1356	5.82	50451
Bp09g1187.1	H	chr09	435	1305	8.7	49515
Bp09g1189.1	H	chr09	467	1401	6.92	52548
Bp04g0795.1	H	chr04	718	2154	6.39	80618
Bp04g0797.1	H	chr04	395	1185	8.45	45043
Bp10g1287.1	I	chr10	459	1377	6.51	51151
Bp10g1288.1	I	chr10	443	1329	5.29	49518
Bp10g1289.1	I	chr10	477	1431	5.05	53358
Bp09g1023.1	J	chr09	460	1380	6.43	52040
Bp09g1021.1	J	chr09	470	1410	5.15	52539
Bp09g1022.1	J	chr09	469	1407	5.74	52882
Bp09g1024.1	J	chr09	461	1383	6.51	51326
Bp09g1025.1	J	chr09	478	1434	6.24	53481
Bp09g1026.1	J	chr09	450	1350	6.37	50563
Bp09g1523.1	K	chr09	472	1416	6.28	53158
Bp04g1740.1	K	chr04	495	1485	6.28	55547
Bp04g1741.1	K	chr04	874	2622	5.84	97898
Bp04g1748.1	K	chr04	478	1434	6.36	53755
Bp04g1749.1	K	chr04	453	1359	5.59	51338
Bp04g1756.1	K	chr04	833	2499	5.1	93792
Bp04g1758.1	K	chr04	473	1419	6.3	52834
Bp12g0701.1	L	chr12	471	1413	5.38	52095
Bp07g0465.1	L	chr07	444	1332	7.13	49434
Bp08g0037.1	L	chr08	460	1380	5.15	50959
Bp08g0038.1	L	chr08	467	1401	5.34	51992
Bp11g1248.1	L	chr11	477	1431	6.47	52546
Bp08g0035.1	L	chr08	453	1359	6	51047
Bp08g1840.1	L	chr08	455	1365	5.75	51712
Bp11g0777.1	L	chr11	470	1410	6.29	52639
Bp11g0778.1	L	chr11	468	1404	5.7	52471
Bp11g0779.1	L	chr11	238	714	6.54	26469
Bp11g0780.1	L	chr11	451	1353	7.58	51094
Bp13g0383.1	L	chr13	471	1413	5.11	52647
Bp13g0384.1	L	chr13	485	1455	5.06	53919
Bp13g0385.1	L	chr13	446	1338	5.48	49811
Bp13g0386.1	L	chr13	480	1440	5.78	53266
Bp09g1998.1	M	chr09	501	1503	6.5	55838
Bp09g1999.1	M	chr09	491	1473	5.91	55287
Bp09g2000.1	M	chr09	499	1497	6.88	55611
Bp0370013.1	M	scaffold_037	503	1509	6.01	56341
Bp13g0117.1	M	chr13	484	1452	8.21	54569

**Bp10g0392.1**	**N**	**chr10**	**470**	**1410**	**6.87**	**52590**
**Bp06g1001.1**	**O**	**chr06**	**505**	**1515**	**6.05**	**55506**
**Bp2810002.1**	**O**	**scaffold_281**	**505**	**1515**	**6.12**	**55418**

Table S2: The identified gene cluster and duplication analysis of *BpUGT* genes.

Tandem duplication pair	Tandem genes	Chr	Subfamily	Similarity
Pair 1	Bp01g1288	chr01	G	90.43
	Bp01g1289		G	
Pair 2	Bp01g1289	chr01	G	88.51
	Bp01g1290		G	
Pair 3	Bp02g2439	chr02	G	90.62
	Bp02g2440		G	
Pair4	Bp03g0082	chr03	A	83.05
	Bp03g0083		A	
Pair 5	Bp03g0161	chr03	E	99.39
	Bp03g0162		E	
Pair 6	Bp03g0972	chr03	G	70.04
	Bp03g0973		G	
Pair 7	Bp03g0973	chr03	G	75.96
	Bp03g0974		G	
Pair 8	Bp06g0244	chr06	F	94.87
	Bp06g0245		F	
Pair 9	Bp06g0245	chr06	F	93.16
	Bp06g0246		F	
Pair 10	Bp06g0246	chr06	F	76.23
	Bp06g0247		F	
Pair 11	Bp06g0409	chr06	E	82.17
	Bp06g0410		E	
Pair 12	Bp08g0037	chr08	L	89.87
	Bp08g0038		L	
Pair 13	Bp08g2158	chr08	E	73.64
	Bp08g2159		E	
Pair 14	Bp09g1021	chr09	J	79.96
	Bp09g1022		J	
Pair 15	Bp09g1185	chr09	H	85.43
	Bp09g1186		H	
Pair 16	Bp09g1186	chr09	H	77.58
	Bp09g1187		H	
Pair 17	Bp09g1998	chr09	M	75.95
	Bp09g1999		M	
Pair 18	Bp10g0376	chr10	G	92.11
	Bp10g0377		G	
Pair 19	Bp10g0379	chr10	G	81.22
	Bp10g0380		G	
Pair 20	Bp10g0380	chr10	G	82.66
	Bp10g0381		G	
Pair 21	Bp10g0416	chr10	E	73.96
	Bp10g0417		E	
Pair 22	Bp10g0419	chr10	E	74.59

	Bp10g0420		E	
Pair 23	Bp10g0690	chr10	A	72.44
	Bp10g0691		A	
Pair 24	Bp10g1288	chr10	I	82.16
	Bp10g1289		I	
Pair 25	Bp11g0777	chr11	L	82.03
	Bp11g0778		L	
Pair 26	Bp11g0778	chr11	L	84.78
	Bp11g0779		L	
Pair 27	Bp13g0383	chr13	L	90.4
	Bp13g0384		L	
Pair 28	Bp13g0385	chr13	L	92.62
	Bp13g0386		L	

Table S3: ***Cis*****-element analysis of 2,000 bp nucleotide sequences data upstream of the translation initiation codon of** ***BpUGT*** **genes.**

The functional description of putative cis-acting elements identified in the promoter regions of BpUGT genes.			numbers
Light reponsive elements	3-AF1 binding site	light responsive element	14
	ACA-motif	part of gapA in (gapA-CMA1) involved with light responsiveness	3
	ACE	cis-acting element involved in light responsiveness	24
	AE-box	part of a module for light response	48
	AT1-motif	part of a light responsive module	25
	ATC-motif	part of a conserved DNA module involved in light responsiveness	15
	ATCT-motif	part of a conserved DNA module involved in light responsiveness	18
	Box 4	part of a conserved DNA module involved in light responsiveness	140
	Box II	part of a light responsive element	10
	CAG-motif	part of a light response element	3
	chs-CMA1a	part of a light responsive element	26
	chs-CMA2a	part of a light responsive element	14
	chs-Unit 1 m1	part of a light responsive element	5
	GA-motif	part of a light responsive element	26
	Gap-box	part of a light responsive element	11
	GATA-motif	part of a light responsive element	70
	GATT-motif	part of a light responsive element	1
	G-Box	cis-acting regulatory element involved in light responsiveness	137
	GT1-motif	light responsive element	80
	GTGGC-motif	part of a light responsive element	3
	I-box	part of a light responsive element	45

	LAMP-element	part of a light responsive element	13
	L-box	part of a light responsive element	4
	MRE	MYB binding site involved in light responsiveness	35
	Sp1	light responsive element	16
	TCCC-motif	part of a light responsive element	35
	TCT-motif	part of a light responsive element	90
Hormone reponsive elements	ABRE	cis-acting element involved in the abscisic acid responsiveness	136
	AuxRE	part of an auxin-responsive element	3
	AuxRR-core	cis-acting regulatory element involved in auxin responsiveness	19
	CGTCA-motif	cis-acting regulatory element involved in the MeJA-responsiveness	117
	ERE	ethylene-responsive element	118
	GARE-motif	gibberellin-responsive element	30
	P-box	gibberellin-responsive element	48
	SARE	cis-acting element involved in salicylic acid responsiveness	2
	TATC-box	cis-acting element involved in gibberellin-responsiveness	27
	TCA-element	cis-acting element involved in salicylic acid responsiveness	76
	TGA-box	part of an auxin-responsive element	11
	TGA-element	auxin-responsive element	58
	TGACG-motif	cis-acting regulatory element involved in the MeJA-responsiveness	117
Development related elements	AACA-motif	involved in endosperm-specific negative expression	2
	AC-I	cis-acting regulatory element related to meristem specific activation	10
	AC-II	cis-acting regulatory element related to meristem specific activation	2
	CAT-box	cis-acting regulatory element related to meristem expression	56
	CCGTCC-box	cis-acting regulatory element related to meristem specific activation	33
	circadian	cis-acting regulatory element involved in circadian control	29
	GCN4_motif	cis-regulatory element involved in endosperm expression	18
	HD-Zip 1	element involved in differentiation of the palisade mesophyll cells	10
	MBSI	MYB binding site involved in flavonoid biosynthetic genes regulation	9
	MSA-like	cis-acting element involved in cell cycle regulation	10
	O2-site	cis-acting regulatory element involved in zein metabolism regulation	55
	RY-element	cis-acting regulatory element involved in seed-specific regulation	2

**Environmental stress-related elements**	**ARE**	**cis-acting regulatory element essential for the anaerobic induction**	**127**
	**DRE**	**cis-acting element involved in dehydration, low-temp, salt stresses**	**1**
	**GC-motif**	**enhancer-like element involved in anoxic specific inducibility**	**8**
	**LTR**	**cis-acting element involved in low-temperature responsiveness**	**48**
	**MBS**	**MYB binding site involved in drought-inducibility**	**72**
	**TC-rich repeats**	**cis-acting element involved in defense and stress responsiveness**	**47**
	**W box**	**cis-acting element involved in sugar metabolism and plant defense signaling**	**58**
	**WUN-motif**	**wound-responsive element**	**68**

Table S4. Expression profiles for *BpUGTs* across different tissues.

	**F**	**LA**	**LB**	**LC**	**RA**	**RB**	**SA**	**SB**	**SC**	**SD**
Bp01g1596	0	0.028355	0.076193	0	0.08434	0.020226	0.022809	0	0.076705	0.208916
Bp3810001	1.753435	1.867322	1.586629	1.499406	3.897186	1.551889	1.592817	1.716162	2.05332	1.755358
Bp10g0689	6.515888	5.134418	4.032044	5.929008	4.684804	3.049999	6.334528	6.033394	1.161873	0.649325
Bp10g0690	3.268257	3.812461	4.032921	6.707615	2.447154	4.338744	4.775317	4.141558	2.314755	1.316353
Bp10g0691	0.454519	0.728479	0.728063	1.438817	4.442544	3.843294	0.598421	1.412072	1.709007	1.275889
Bp04g0926	0	0	0	0	0	0	0	0	0	0.126177
Bp03g0083	0	0	0.01932	0	0	0	0	0	0	0
Bp03g0082	0	0	0.023936	0	0	0	0	0	0.022908	0.090546
Bp13g0883	0	0.204671	0.263438	0.089819	4.785447	21.96705	0.226669	0.367325	1.844441	3.831371
Bp01g0501	14.9056	8.932285	1.684498	0.693199	0.537404	1.403332	21.44433	4.20718	1.114648	1.918039
Bp01g0502	5.40437	1.19511	1.681476	2.013435	32.2352	14.81357	1.73284	7.044411	24.3476	8.96256
Bp02g1287	0	0	0	0	0	0	0	0	0	0
Bp03g1557	0	0	0	0	0	0.05528	0	0	0.022713	0
Bp0760001	9.980236	16.35412	9.696362	8.086036	8.012847	4.437977	12.48582	11.72507	19.42731	12.06519
Bp12g1069	2.025311	3.756755	1.489427	0.155587	7.64774	1.411562	6.790904	10.39203	11.15342	1.282535
Bp12g1070	5.338477	1.450122	1.729045	1.46202	0.549912	0.11305	0.736012	0.417925	0.955266	0.922237
Bp07g0795	3.04345	4.747352	3.419265	3.122597	4.003637	4.370155	3.313932	6.411756	10.3943	11.26435
Bp02g1046	0.097433	1.079807	0.139476	0.058649	4.856442	12.97548	0.889042	0.081724	0.070509	0.115321
Bp1250006	9.36377	2.154975	3.973068	5.470151	0.949932	4.391243	2.744346	4.924165	4.791236	13.05269
Bp11g0478	0.96255	0.167022	0.298254	1.788175	0.504953	1.472259	0.152202	0.051752	1.031614	1.302983
Bp11g0480	3.217553	6.691165	7.453098	14.0899	7.799379	6.050086	4.642286	2.386146	5.401274	15.50247
Bp11g0481	0.079782	0.028803	0.001386	0.076425	0	0.043296	0.021024	0.026759	0.144545	0.132171
Bp11g0482	0	0	0.006977	0	0	0	0.024745	0	0.017578	0.042308
Bp11g0484	2.278306	0.811926	0.667289	1.434647	6.645019	3.596252	1.678787	2.057186	1.779975	2.253653
Bp11g0485	0	0.832985	0.252675	0.070088	6.625391	4.798938	1.038043	1.648456	2.241237	0.174892
Bp06g1330	0	0	0	0	0.274311	0.273863	0	0	0.01223	0.078721
Bp06g1332	0.077051	0.051329	0.036722	0.059282	3.27714	4.480706	0.031501	0.148679	0.149558	2.778075
Bp2800001	0.645819	0.745441	0.833233	1.084574	4.817332	3.50707	1.382936	2.085766	2.383984	1.951676
Bp2800003	0	0	0	0	0	0	0	0	0	0
Bp11g0476	8.868897	6.766988	5.659718	4.327549	18.79161	5.694316	7.095876	8.116361	3.099545	6.53529
Bp11g0475	0	0	0	0	0	0	0	0	0	0
Bp11g0477	0.070434	0.712185	0.859204	1.038923	0.012481	0.505408	1.398591	0.644567	0.602122	0.060594
Bp11g0473	0.843047	3.878921	2.144053	0.844493	0	0	0.683298	2.91054	0.446339	0.218278

Bp01g2416	2.034227	0.708668	1.29805	7.988997	0.555687	1.577107	0.459797	0.224066	1.138721	0.599689
Bp04g1633	0.039307	0.049666	0.150484	0.372045	0.056567	0.07108	0.183924	0	0.072843	0.363065
Bp04g1634	0.228811	1.914325	3.562973	2.910565	0	0.06303	0.895155	1.419274	0.722803	0.324406
Bp04g1654	2.331531	2.902203	1.093076	0.820908	0.535026	1.208476	2.259919	0.689493	2.075317	2.434498
Bp04g1655	0.160768	0.163214	0.125874	0.086605	0.299833	0.58078	0.388594	0.370809	0.494944	2.466894
Bp04g1656	0.328632	1.243571	0.219689	0.31552	0.539287	0.154736	1.426157	0.55643	0.88517	2.447601
Bp09g1107	0.018961	11.04843	8.878663	8.107885	2.781634	0.075011	6.952896	3.189201	0.08741	0.10754
Bp09g1108	0.49097	0.846055	1.285638	1.04317	1.418982	0.179492	1.003615	2.853138	0.367281	0.312833
Bp09g1109	5.492428	26.25078	23.08486	17.46903	12.54708	6.179973	13.79381	9.37777	9.138006	13.54864
Bp09g1111	0.996757	1.542224	3.988503	0.710801	0.763865	0.277473	0.877349	1.201463	0.328566	0.425417
Bp09g1112	2.101532	41.4597	45.0542	21.6895	0.046148	0.013752	17.70834	7.726088	0.703164	0.372599
Bp09g1115	8.360898	5.167766	4.140464	2.073297	15.68496	12.12613	8.68803	13.17371	7.191738	7.758443
Bp09g1117	0.435715	7.436524	6.590617	5.454984	5.737054	2.450239	4.433085	12.68342	4.600381	4.089187
Bp4920002	0	0	0	0	0	0	0	0	0	0
Bp10g0424	0	0	0	0	0	0	0	0	0	0
Bp10g0416	8.112048	20.43346	9.174978	11.29011	0.209323	0.262061	19.9886	1.713878	3.360399	7.172279
Bp10g0417	1.386457	13.90831	8.071807	29.0267	11.14581	6.809621	12.31515	11.20608	12.67724	17.97152
Bp10g0418	3.740869	0.644557	0.326134	0.851471	13.78777	21.22644	0.826604	4.292263	4.097842	10.02738
Bp10g0419	4.956109	8.550711	3.014408	6.888471	14.87781	8.049707	6.539036	9.215618	6.542137	16.3249
Bp10g0420	3.943329	10.11843	6.296134	2.80387	26.55788	11.12006	9.333505	5.722988	5.017853	23.80937
Bp08g2158	0.230541	0.029946	0.177022	0.431751	1.792733	0.522233	0.164568	0.18823	0.095793	0.52641
Bp08g2159	0.713081	0.069078	0	0.061328	2.310136	0.616139	0.249026	0.040992	0.037936	0.282648
Bp03g0350	0	0	0	0	0	0	0	0	0	0
Bp03g0351	0	0	0.167189	0.359706	5.064232	1.704226	0.386301	0	18.46501	27.74354
Bp03g0353	0	0.135194	0.564306	2.279277	8.374386	3.42185	0.123916	20.3498	4.41484	2.198645
Bp03g0354	0	0	0.016358	0.015217	0	0	0.023119	0.077352	0.017697	0.199166
Bp03g0160	3.015918	2.341904	2.412562	2.498184	5.838424	6.117842	3.888769	2.202255	2.06511	7.700517
Bp09g2125	0	0	0	0	0	0	0	0	0	0
Bp05g1012	0.70912	17.10853	14.39152	10.19615	35.95196	30.95041	9.229591	39.56965	105.439	48.02916
Bp01g0865	0	0	0	0	0	0	0	0	0	0
Bp01g0136	106.4131	3.997756	2.570482	1.525625	17.78905	29.38778	12.50142	25.79674	11.40295	22.79979
Bp06g0409	2.475621	0.000932	2.191207	12.91485	0.001805	0.000659	0.002077	0.002437	0	0.001154
Bp06g0410	7.956469	14.12623	12.53016	22.57076	1.690948	0.764108	9.345113	13.9577	5.008131	3.434887
Bp06g0411	0.032531	0.093289	0.098345	0.048631	0	0	0.058501	0.031201	0.067284	0.432805
Bp01g2634	4.659041	6.571613	1.510378	2.736516	4.923181	2.567541	7.64632	3.235055	2.463069	1.030853
Bp01g0137	0	0.023301	0.031458	0	1.617301	0.31727	0.038019	0	0.112607	0.924194
Bp01g0139	0	0.012937	0.023	0	0.011828	0.046381	0.020931	0	0	0.096713
Bp04g0924	0	0.026777	0.023047	0.013113	0.119309	0	0.097874	0.012725	0	0.281277
Bp07g0695	17.75127	16.39865	16.56412	5.605015	4.141457	2.046236	14.03855	8.605938	6.152411	1.220842
Bp03g0161	0	0.014462	0.014015	0.062212	0.871721	0.442417	0.080684	0.207485	0.02893	0.255585
Bp03g0162	0.026766	0.174306	0.214123	0.155898	1.780181	1.291375	0.268558	0.591283	0.035592	0.55975
Bp10g1327	25.70023	29.7073	17.97616	6.031082	12.04539	4.759264	23.74199	4.411917	5.644058	7.286794
Bp10g1329	0	0	0	0	0	0	0	0	0	0
Bp04g0989	1.42345	0.382938	1.477534	1.383619	3.63012	2.643241	0.500946	0.159799	0.509833	0.411822
Bp04g0991	0	0.076048	0.156281	0	1.321181	0.024403	0.149093	0.171891	0.025316	0.171976
Bp04g0993	0	0.026781	0	0	0.114595	0	0	0	0.022149	0.150621
Bp04g0996	0	0.041271	0	0	0	0	0	0	0	0.035304
Bp1420001	13.15496	24.99085	23.05874	17.87291	0.764003	0.485401	14.82487	12.46821	11.1264	2.35365
Bp2520001	6.176571	11.85846	11.89366	8.849946	0.574503	0.267593	7.742813	5.656916	4.528558	1.009292
Bp04g0971	6.732577	3.376625	5.173756	11.83393	2.247152	2.82095	6.255644	1.219663	0.702132	2.020221
Bp04g0973	0.565059	0.561278	0.224497	0.243558	3.8157	3.089612	1.312806	0.952607	0.06746	0.562498
Bp04g0987	2.756357	0.630645	0.564366	1.010673	51.04014	42.80709	1.6588	1.351184	3.537589	10.1738

Bp03g0068	21.0362	22.13023	15.03939	16.11489	0.712216	0.057604	12.1703	7.765141	1.226731	0.320541
Bp05g1474	0.327983	0.114195	0.215277	0	0	0	0	0	0	0.035417
Bp06g0244	0	0.02051	0	0	5.61523	2.464005	0	0.019779	0.525169	10.39654
Bp06g0245	0.170657	0.264061	0.2408	0.033702	7.918029	11.57955	0.138032	0.338512	4.113823	20.81742
Bp06g0246	0	0.058467	0	0	0.43023	0.833978	0.011455	0.247019	3.109464	12.75438
Bp06g0247	10.64207	92.20547	89.81859	59.65518	0.041121	0	52.71198	3.436501	0.424583	0.400726
Bp03g0972	0.812288	0.071339	0.175916	0.222026	12.08671	3.071214	0.163642	1.341611	1.595509	1.543856
Bp03g0973	0	0.013306	0.050359	0.046617	0.098159	0.024584	0.034665	0	0	0.262169
Bp03g0974	9.215951	1.531125	6.663439	15.92541	0	0.020737	1.593575	1.835761	1.710746	1.970593
Bp03g0979	0	0.033633	0.116083	0	0	0	0.072201	0	0.047223	0.356873
Bp02g2438	8.869067	8.51429	3.091955	0.697159	1.397928	4.959547	13.81508	12.4311	4.751251	1.140467
Bp02g2439	4.423212	16.90752	6.049002	0.597371	0.661389	0	11.19996	0.146239	0.101866	0.627804
Bp02g2440	0	0.141545	0.16468	0.165849	0.173277	0.103642	0.202929	0.050153	0.119016	0.890014
Bp02g2441	0	0	0.064425	0.020211	0.492375	0.7463	0.030255	0	0.021833	0.189665
Bp11g0255	0	0	0.082843	0	0	0	0	0.025248	0.021146	0.114943
Bp01g1288	3.040606	2.491932	8.211111	18.10809	3.807909	3.329553	1.6907	0.328441	0.16368	0.045721
Bp01g1289	0.665904	1.036297	0.693044	1.798185	11.41556	13.03408	1.686327	0.742033	1.130801	6.580124
Bp01g1290	3.599017	4.388552	11.9729	16.95807	52.39471	67.59205	2.424347	2.485678	1.53078	2.389648
Bp01g1292	0	0.372118	0.065069	0.78148	0	0	0.161322	0.172605	0.102391	0.264838
Bp10g0376	0	0.679175	1.102623	0.351177	1.449497	0.386511	0.719824	0.74561	0.820109	0.921396
Bp10g0377	0.037898	1.831447	2.140131	0.12496	10.07514	3.292663	0.131814	2.140685	3.52634	3.850586
Bp10g0379	8.303251	3.332353	3.608851	4.080923	4.339069	6.530333	3.220542	3.361373	4.404992	4.69666
Bp10g0380	7.966356	3.989401	4.727361	4.091572	16.27176	17.30757	4.020871	2.093718	1.800009	19.76755
Bp10g0381	2.101173	4.839684	14.70001	20.27309	0.206883	0.098913	2.696792	3.889752	2.174582	0.925089
Bp12g0355	7.772379	2.06966	0.764127	1.083292	2.262788	1.573065	3.144456	1.501742	3.023059	3.837735
Bp09g1185	0	0.143179	0.120564	0.354486	0.388564	0.622161	0.119847	0.083057	0.18717	2.514895
Bp09g1186	4.096148	2.307548	3.834289	11.06745	29.1621	16.6529	1.903778	2.206382	0.641845	7.775137
Bp09g1187	1.544187	2.536084	2.083788	4.890115	4.478303	3.219788	2.680582	1.276273	1.25763	2.311329
Bp09g1189	0.666138	0.305935	0.958983	6.219173	0.233092	0.029751	0.290976	0.073873	0.090992	0.060746
Bp04g0795	2.510769	12.62022	7.828392	3.782663	8.223686	16.50875	7.321425	6.504383	18.71279	21.0607
Bp04g0797	0	0	0	0	0.024367	0	0	0.041944	0.071535	0.096648
Bp10g1287	34.21143	0.540348	0.087856	0.110206	3.002915	0.237879	1.481	0.887064	0.414027	1.751955
Bp10g1288	0.083826	0.034975	0.167844	0.109811	0.865565	0.07743	0.071885	0.057856	0.227933	0.592202
Bp10g1289	0	0	0.005475	0.0185	0.095949	0.127755	0.092634	0.002559	0.01119	0.142329
Bp09g1023	0.371626	0.989308	0.514596	0.33405	0.648312	0.530442	0.868733	0.109781	0.206071	0.809633
Bp09g1021	3.693907	2.287932	1.313724	1.199574	6.426235	7.835654	3.364646	2.592574	4.908297	7.298642
Bp09g1022	0	0	0	0	0	0.000916	0	0	0	0
Bp09g1024	18.32838	28.65223	42.45526	28.18171	8.680622	12.3917	24.41291	16.03535	19.8118	24.17283
Bp09g1025	0.967066	3.094196	1.460016	1.388902	12.0504	19.15024	3.011541	3.271111	4.903513	14.64752
Bp09g1026	12.97514	6.355454	7.127984	16.47059	18.36638	13.41019	7.120389	14.37292	11.5761	16.80908
Bp09g1523	0	0.051766	0.039316	0.052943	0.040833	0	0.070841	0.024537	0.067404	0.555254
Bp04g1740	0.630345	0.748483	0.232762	0.706155	0.539018	1.597774	0.272016	0.110514	0.017673	0.065765
Bp04g1741	0.059656	0.289397	0.249269	2.557729	0.387883	1.184168	0.069492	0.028989	0.335518	6.457698
Bp04g1748	0.080912	0	0.36793	1.054843	0	0.012473	0.011546	0.026708	0.101143	0.181971
Bp04g1749	0	0	0.014372	0.026047	0.423969	0.515407	0.025079	0.020601	0.046303	0.064754
Bp04g1756	1.032588	0.443994	0.797426	1.658745	0.334036	0.062606	0.352965	0.269756	0.095623	0.143459
Bp04g1758	46.51022	20.86202	15.55798	26.84122	1.764618	1.006432	15.80101	21.15252	3.244317	4.552124
Bp12g0701	0.531955	1.075351	1.326771	1.826635	9.636666	6.682974	1.098722	1.31144	1.591169	3.879088
Bp07g0465	1.626871	13.44066	20.46428	29.24283	4.192549	0.676416	4.758971	3.243924	0.188286	1.507393
Bp08g0037	0.681464	2.355072	0.863833	0.232054	0.770604	0	2.740974	0.364526	0.061902	0.104069
Bp08g0038	2.382902	3.058722	3.104956	1.960419	0.181083	0	2.168863	1.589059	0.530783	0.071352
Bp11g1248	0	2.547168	1.397624	0.490578	0.235097	1.080757	3.529378	2.654006	3.288204	0.458036

Bp08g0035	1.686609	0.529493	3.460847	4.638936	0.028046	0	1.010389	0	0.08897	0.279156
Bp08g1840	5.638554	6.930854	6.777403	10.90633	5.514901	4.827028	7.508111	5.3343	4.270934	6.545964
Bp11g0777	0.236001	1.86192	3.322518	0.950832	0.685355	0.127782	0.57714	0.153639	0.468494	0.78345
Bp11g0778	0.870652	0.246156	4.678538	5.551011	3.046708	3.234983	0.928656	0.493236	1.146082	2.93586
Bp11g0779	1.559748	0.796885	2.143676	4.555589	1.398801	2.281926	0.499352	1.516803	1.634923	3.127202
Bp11g0780	24.60848	14.25797	29.24622	24.81498	7.385873	17.05227	10.49275	13.82994	15.99632	18.38719
Bp13g0383	4.32948	10.71359	10.95777	4.633125	5.157465	5.409653	5.215991	2.321068	2.777889	7.447287
Bp13g0384	2.479172	18.01599	13.64162	14.96054	3.862757	5.671233	4.853097	2.951668	2.695475	8.927376
Bp13g0385	0.753703	0.850231	0.965061	0.255671	1.779488	0.28368	2.66465	0.295065	0.141372	0.244901
Bp13g0386	0.716812	0.896434	0.707998	1.601784	3.400219	3.472028	1.326675	0.33315	0.84142	5.045026
Bp09g1998	0	3.226181	1.220007	0.226741	1.132743	5.641978	2.815393	6.475062	19.90256	18.99846
Bp09g1999	0	2.170149	0.488832	0	0.426625	1.535356	1.756631	2.899929	7.323855	5.290188
Bp09g2000	10.08244	30.98256	20.3294	6.690678	32.63506	12.96945	19.10665	21.84209	43.35382	21.53623
Bp0370013	32.39314	12.06987	15.18627	14.05073	13.06377	10.76886	13.17068	20.72147	14.14734	14.25276
Bp13g0117	7.459461	1.05805	2.13675	2.515802	0.310437	10.33632	0.974484	1.313656	0.058747	0.509472
Bp06g1001	2.472656	2.060009	3.502987	4.332213	14.24876	23.01254	1.737058	9.616898	34.13235	14.46708
Bp2810002	2.060143	1.805874	3.303106	4.742633	14.67233	14.68158	1.818991	10.16072	32.7596	14.92863
Bp10g0392	0.466653	0.65184	1.080132	3.284624	1.326463	1.125809	1.526341	6.470307	8.352774	6.235792

Table S5. Quantitative results of detected compounds in *B. kazinoki*, *B. papyrifera* and *B. kazinoki* × *B. papyrifera* leaves.

Peak	Compound	*B. kazinoki*	*B. papyrifera*	*B. kazinoki* × *B. papyrifera*
1	Neochlorogenic acid	1467.2±97.7	5477.5±60.6	6490.6±61.1
2	Chlorogenic acid	1523.7±90.4	1572.6±96.5	4384.2±44.2
3	Di-*C*, *C*-hexosyl-luteolin	222.4±10.3	6.3±0.9	122.0±7.5
4	Di-*C*, *C*-hexosyl-apigenin	1088.5±29.5	43.5±11.4	782.7±11.0
5	Isoorientin	17.3±0.8	256.8±2.6	388.0±19.7
6	Vitexin-7-*O*-β-D-glucopyranoside	ND	133.2±7.8	448.3±13.4
7	Orientin	4.3±0.3	633.4±31.9	319.0±10.9
8	Schaftoside	ND	132.2±5.9	153.4±4.2
9	5,7,4'-trihydroxyl-6-*C*-Rhamnopyranosyl-β-D-glucopyranosyl flavone	ND	315.6±15.5	230.6±5.8
10	Isoschaftoside	ND	117.7±1.2	292.0±0.8
11	Vitexin	123.1±3.149	1629.329±53.36	1336.0±29.0
12	5,7,4'-trihydroxyl-8-*C*-Rhamnopyranosyl-β-D-glucopyranosyl flavone	ND	21.7±1.0	19.7±1.9
13	Luteolin-7-*O*-β-D-glucopyranoside	211.6±1.9	184.529±2.7	987.2±12.1
14	Luteolin-7-*O*-β-D-glucopyranuronide	1535.7±26.1	1203.49±22.2	1779.2±19.5
15	Apigenin-7-*O*-β-D-glucopyranoside	140.4±1.8	96.2±2.0	991.7±28.7
16	Apigenin-7-*O*-β-D-glucopyranuronide	2335.4±24.3	1837.8±49.2	2422.3±15.6
17	Dihydroapigenin derivative	370.0±10.4	13.0±1.0	77.9±2.3
18	Luteolin	2.6±0.1	3.4±0.4	27.4±2.4

19	Apigenin	2.2±0.1	ND	9.2±0.6

ND: not detected.
